# Supplementary material for: Trimester-Specific Serum Fructosamine in Association with Abdominal Adiposity, Insulin Resistance, and Inflammation in Healthy Pregnant Individuals
Source: Nutrients. 2022 Sep 27;14(19):3999. doi: 10.3390/nu14193999 (PMC9572673; doi:10.3390/nu14193999)
Supplement: Supplementary file 1 [file nutrients-14-03999-s001.zip › nutrients-1904692-supplementary.pdf]

Supplementary Materials

Table S1. Trimester-specific metabolic markers data, according to pre-pregnancy BMI categories

		First trimester		Second trimester		Third trimester	
		n	Mean ± SD	n	Mean ± SD	n	Mean ± SD
**ppBMI < 25 kg/m²**	*Adiposity markers*						
	ppBMI (kg/m²)	58	22.0 ± 1.9	-	-	-	-
	SAT thickness (mm)	53	12.9 ± 4.9	-	-	-	-
	VAT thickness (mm)	53	20.4 ± 0.1	-	-	-	-
	Leptin (ng/ml)	44	20.9 ± 10.8	44	24.5 ± 11.7	44	26.0 ± 14.5
	Glucose homeostasis markers						
	Fasting glucose (mmol/L)	58	4.4 ± 0.3	57	4.4 ± 0.4	58	4.5 ± 0.4
	Fasting insulin (pmol/L)	56	49.9 ± 21.6	55	58.6 ± 21.1	56	77.2 ± 26.8
	HOMA-IR	56	1.4 ± 0.6	55	1.7 ± 0.7	56	2.2 ± 0.9
	Inflammation markers						
	Adiponectin (mg/ml)	42	11.4 ± 2.8	42	10.2 ± 3.1	42	8.3 ± 2.8
	IL-6 (pg/ml)	39	0.8 ± 0.5	39	1.1 ± 1.0	39	1.4 ± 0.8
	CRP (mg/L)	42	5.6 ± 7.3	42	4.5 ± 3.7	42	4.8 ± 5.8
**25 < ppBMI < 30 kg/m²**	*Adiposity markers*						
	ppBMI (kg/m²)	25	26.8 ± 1.4	-	-	-	-
	SAT thickness (mm)	24	19.6 ± 5.6	-	-	-	-
	VAT thickness (mm)	24	29.5 ± 10.3	-	-	-	-
	Leptin (ng/ml)	19	43.2 ± 14.4	19	43.1 ± 13.4	19	47.2 ± 16.2
	Glucose homeostasis markers						
	Fasting glucose (mmol/L)	25	4.6 ± 0.4	25	4.5 ± 0.4	24	4.8 ± 0.7
	Fasting insulin (pmol/L)	24	63.9 ± 19.0	23	79.6 ± 21.5	21	103.7 ± 32.4
	HOMA-IR	24	1.9 ± 0.6	23	2.3 ± 0.7	21	3.1 ± 1.1
	Inflammation markers						
	Adiponectin (mg/ml)	19	8.8 ± 2.8	19	8.4 ± 3.0	19	7.2 ± 2.7
	IL-6 (pg/ml)	18	1.1 ± 0.6	18	1.0 ± 0.5	18	1.5 ± 0.8
	CRP	19	7.5 ± 7.7	19	6.24 ± 3.4	19	8.0 ± 6.1
**ppBMI > 30 kg/m²**	*Adiposity markers*						
	ppBMI (kg/m²)	18	35.0 ± 4.7	-	-	-	-
	SAT thickness (mm)	18	27.5 ± 5.7	-	-	-	-
	VAT thickness (mm)	18	43.2 ± 19.3	-	-	-	-
	Leptin (ng/ml)	14	60.4 ± 15.8	14	78.3 ± 36.9	14	81.1 ± 24.2
	Glucose homeostasis markers						
	Fasting glucose (mmol/L)	18	4.6 ± 0.4	18	4.6 ± 0.4	18	4.8 ± 0.4
	Fasting insulin (pmol/L)	17	105.4 ± 48.2	18	118.1 ± 68.6	18	153.1 ± 66.5
	HOMA-IR	17	3.1 ± 1.6	18	3.6 ± 2.3	18	4.8 ± 2.3
	Inflammation markers						
	Adiponectin (mg/ml)	14	8.8 ± 3.5	14	7.2 ± 2.4	14	7.0 ± 2.2
	IL-6 (pg/ml)	15	1.5 ± 0.6	15	1.8 ± 0.8	15	1.8 ± 1.3
	CRP (mg/L)	12	11.2 ± 6.1	12	13.8 ± 7.6	12	10.1 ± 4.3

PpBMI, pre-pregnancy body mass index; SAT, subcutaneous adipose tissue; VAT, visceral adipose tissue; HOMA-IR, homeostasis model assessment of insulin resistance; IL-6, interleukin-6; CRP, C-reactive protein.
